# Abnormalities in Osteoclastogenesis and Decreased Tumorigenesis in Mice Deficient for Ovarian Cancer G Protein-Coupled Receptor 1

**DOI:** 10.1371/journal.pone.0005705

**Published:** 2009-05-28

**Authors:** Hui Li, Dongmei Wang, Lisam Shanjukumar Singh, Michael Berk, Haiyan Tan, Zhenwen Zhao, Rosemary Steinmetz, Kashif Kirmani, Gang Wei, Yan Xu

**Affiliations:** 1 Department of Obstetrics and Gynecology, Indiana University Cancer Center, Indiana University School of Medicine, Indianapolis, Indiana, United States of America; 2 Department of Cancer Biology, Lerner Research Institute, Cleveland Clinic, Cleveland, Ohio, United States of America; 3 Department of Biotechnology, Manipur University, Canchipur, Manipur, India; Health Canada, Canada

## Abstract

Ovarian cancer G protein-coupled receptor 1 (OGR1) has been shown to be a proton sensing receptor *in vitro*. We have shown that OGR1 functions as a tumor metastasis suppressor gene when it is over-expressed in human prostate cancer cells *in vivo*. To examine the physiological functions of OGR1, we generated conditional OGR1 deficient mice by homologous recombination. OGR1 deficient mice were viable and upon gross-inspection appeared normal. Consistent with *in vitro* studies showing that OGR1 is involved in osteoclastogenesis, reduced osteoclasts were detected in OGR1 deficient mice. A pH-dependent osteoclasts survival effect was also observed. However, overall abnormality in the bones of these animals was not observed. In addition, melanoma cell tumorigenesis was significantly inhibited in OGR1 deficient mice. OGR1 deficient mice in the mixed background produced significantly less peritoneal macrophages when stimulated with thioglycolate. These macrophages also showed altered extracellular ***signal***-regulated kinases (ERK) activation and nitric oxide (NO) production in response to lipopolysaccharide. OGR1-dependent pH responses assessed by cAMP production and cell survival in macrophages or brown fat cells were not observed, presumably due to the presence of other proton sensing receptors in these cells. Our results indicate that OGR1's role in osteoclastogenesis is not strong enough to affect overall bone development and its role in tumorigenesis warrants further investigation. The mice generated can be potentially used for several disease models, including cancers or osteoclast-related diseases.

## Introduction

We have previously cloned OGR1 from an ovarian cancer cell line HEY [1]. Recently, we have shown OGR1 is a novel metastasis suppressor gene for prostate cancer [2]. OGR1 and its subfamily G protein-coupled receptors (GPCRs), GPR4, G2A and T-cell death-associated gene 8 (TDAG8), have been shown to have proton-sensing ability [3], [4], [5], [6], [7], [8], [9]. Many of these proton sensing activities have been identified in cells over-expressing one or more of these GPCRs. More recently, proton sensing activities have been detected in cells from GPR4- and TDAG8-, but not G2A-deficient mice [8], [10], [11].

Mice deficient in G2A, TDAG8, or GPR4 have been generated. These knockout (KO) mice are viable and exhibit different phenotypes. G2A KO mice demonstrate a normal pattern of T and B cell lineage differentiation through young adulthood. However, aged G2A-deficient animals (>1 year) develop secondary lymphoid organ enlargement associated with abnormal expansion of both T and B lymphocytes [12]. In addition, other phenotypes related to bone marrow-derived cells, including, monocytes/endothelial cells and macrophages, as well as hepatic cells have been reported [8], [13], [14], [15], [16], [17]. TDAG8 is transcriptionally up-regulated by glucocorticoids (GCs) and implicated by over-expression studies in psychosine-mediated inhibition of cytokinesis [18], [19]. Although TDAG8 expression resembles the dynamic regulation described for known modulators of GC-induced apoptosis during thymocyte development, it is dispensable for psychosine-induced formation of multinucleated cells. In addition, thymocytes in TDAG8 KO mice show normal apoptosis following *in vivo* and *in vitro* GC treatment [11]. Moreover, acidic extracellular pH does not differentially modulate the susceptibility of TDAG8 wild type (WT) and KO thymocytes to GC-induced apoptosis [11]. However, in thymocytes and splenocytes explanted from receptor-deficient mice, TDAG8, but not G2A, is found to be critical for pH-dependent cAMP production [8]. While this manuscript was in preparation, Mogi *et al* have shown that TDAG8, but not OGR1 is involved in pH-induced inhibitory effect on tumor necrosis factor-alpha (TNF-α) production in macrophages [20]. GPR4 is expressed in most endothelial cells and mediates sphingosylphosphorylcholine (SPC)-induced angiogenic activities (11). GPR4 deficiency leads to partially penetrated vascular abnormalities during development and that this receptor functions in blood vessel pH sensing in an *ex vivo* aortic ring assay [10].

OGR1 has been identified as a strongly up-regulated gene during osteoclastogenesis in *csf1^tl^/csf1^tl^* rats (CSF-deficient rat) treated with macrophage colony-stimulating factor (M-CSF or CSF-1) and in receptor activator of nuclear factor κ B ligand (RANKL, or TRANCE, TNFSF11)-induced osteoclast differentiation *in vitro* [21]. The potential functional involvement of OGR1 in osteoclastogenesis has been demonstrated using an anti-OGR1 antibody and by inhibiting OGR1 with small inhibitory RNA (siRNA) [21]. Moreover, systemic acidosis has detrimental effects on the skeleton and local acidosis is associated with bone destruction in inflammatory and neoplastic diseases. Survival and calcium signaling of osteoclasts are significantly enhanced by acidification of the medium in an OGR1-depedent manner [22]. The functional involvement of OGR1 has mainly been examined using either OGR1 antibody, or a nonspecific OGR1 antagonist Cu^2+^, or siRNAs against OGR1. Since all these reagents all potentially can have off-target effects, a more specific system is needed to assess the physiological roles of OGR1.

Mesenchymal stem cells (MSCs) and hematopoietic stem cells from bone marrow are capable of differentiating into monocytes, osteoclasts, osteoblasts, and adipocytes, among other cell phenotypes. These cell types are important for many biological functions. Under normal physiological conditions, the differentiation processes are tightly controlled. Imbalanced differentiation and/or activation processes lead to pathological conditions and diseases.

Derived from blood monocytes, macrophages are relatively long lived phagocytotic cells of mammalian tissues. Macrophages are involved in a variety of processes including pathogen destruction, inflammation, tissue repairing and remodeling. They have a highly plastic phenotype and their functional polarization is determined by cytokines and factors found within local microenvironments [23]. One of the functions of macrophages is to provide a defense mechanism against tumor cells. However, tumor-associated macrophages (TAMs), which represent the major inflammatory component of the stroma of many solid tumors, are associated with tumor progression and metastasis [23].

Brown and white adipose tissues (BAT and WAT) are key players in obesity and related with health problems, such as type-2 diabetes and cardiovascular diseases. BAT-dependent non-shivering thermogenesis significantly affects the body's energy balance. In addition to its energy storage function, the fat tissues also secrete a number of hormones and cytokines, and are involved in the control of body metabolism and energy balance at multiple sites [24], [25]. BAT is of particular importance in neonates, small mammals (such as mice) in cold environments, and animals that hibernate, because its major physiological function is to dissipate stored energy as heat. In human infants BAT comprises up to 5% of total body weight, which then diminishes with age to virtually nonexistent levels by adulthood.

Throughout an animal's life, bone tissue is in a constant state of turnover as a result of the combination of sequential removal of bone tissue by osteoclasts and new bone deposition by osteoblasts. Osteoclasts are bone-resorbing multinuclear giant cells that are derived from hematopoietic mononuclear precursor cells under the control of both M-CSF and RANKL [21]. These cells aid in absorbing and removing excess bone tissues in the remodeling of growing bones, or damaged bone in the repair of fractures.

We have generated and characterized OGR1-deficient mice to address several critical issues: 1) the physiological functions of OGR1 in mice; 2) the role of OGR1 in osteoclastogenesis; 3) the OGR1-dependent pH responsiveness using OGR1-deficient cells; and 4) the functions of OGR1 in other bone biological processes when mice are challenged by exogenous stimuli, such as tumor cells. We have found that in consistence with published data, OGR1 is likely to be involved in osteoclastogenesis. In our hands, we observed reduced osteoclasts derived from bone marrow cells of OGR1 deficient mice. A weak pH-dependent osteoclasts survival effect was also observed. However, the overall bone structures of the mice were not affected and a significant pH-regulated and OGR1-dependent biological effect was not generally evidenced in OGR1-expressing cells [including macrophages and brown adipose derived cells (BADCs)], suggesting a redundant effect of other OGR1 subfamily GPCRs *in vivo*. In addition, the effect and involvement of host cell OGR1 in tumorigenesis of melanoma cells is of great interest and warrants further investigation. Two additional abnormalities related to peritoneal macrophages and BAT were observed in a mouse background-dependent manner, suggesting involvement of modifying genes in different mouse backgrounds.

## Materials and Methods

### Reagents

Sphingosylphosphorylcholine (SPC) and lysophosphatidylcholine (LPC) were from Aventi Polar Lipids (Birmingham, AL) or Toronto Research Chemicals (Toronto, Canada). M-CSF and RANKL were from Peprotech (Rocky Hill, NJ). Anti-FLAG M2, H_2_O_2_, thioglycolate (TG), lipopolysacchride (LPS), Latex beads, sodium nitrite, Griess reagent, tartrate-resistant acid phosphatase (TRAP) staining reagents and Masson's Trichrome staining reagents were purchased from Sigma (St. Louis, MO**).** Anti-phospho-ERK, anti-ERK, anti-phospho-p38, anti-p38 were from Cell Signaling Technology (Beverly, MA). Goat anti-iNOS, TRITC-anti-rabbit were from BD (San Jose, CA). Anti-F4/80, anti-PCNA, rabbit anit-arginase and anti-cyclooxygenase-2 (COX-2) were from Santa Cruz (Santa Cruz, CA). Anti-CD31 was from RDI (Fitzgerald, Concord, USA). Citrate buffer, universal horse serum, VECTOR NovaRED, and hematoxylin QS were from VECTOR (Burlingame, CA).

### Generation of OGR1 knockout (KO) mice

OGR1 targeting construct has been generated through a contract with AnTeq Transgenic Mice (Australia). In brief, A 13.2 kb Bam HI genomic fragment containing OGR1 gene was subcloned from a bacterial artificial chromosome (BAC) clone (a kind gift from Dr. Owen Witte's lab at UCLA) into a pBS (KS) II vector (plasmid #182). The 5′ homology region of the OGR1 locus was PCR-amplified using OGR1 primers #1 and #2 and plasmid #182 as a template. Primer #2 created a new Nco1 site at the beginning of OGR1 (changed the first codon from ATG to CTC). The PCR fragment was subcloned into pCR2.1.TOPO (Invitrogen) to give plasmid #183. Exon 1 of OGR1 was PCR-amplified using primers #7 and #8, while primer #7 introduced a Xba I site for further cloning steps. The PCR fragment was subcloned into pCR2.1.TOPO to give plasmid #188. This complete OGR1 sequence was confirmed by sequencing analyses. A 6.2 kb genomic fragment of the 3′ untranslated region of the OGR1 locus was subcloned into plasmid #182 to give plasmid #189. A 1.4 kb floxed–neo-cassette was then cloned into plasmid #189 linearized with Stu I to give plasmid #208. Stu I cuts at the 5′ end of the 3′ Hind III OGR1 region. The 3xFLAG region encoding three adjacent FLAG epitopes was PCR-amplified from plasmid p3xFLAG CMV-19 using primers #3 and #4 and the latter primer included an Xho I site for further cloning. The 353 bp PCR product was subcloned into pCR2.1.TOPO (plasmid #185). A 400 bp Ken I and Xho I fragment from #185 was further subcloned in pBS(KS) II to get plasmid #196. A 2.6 kb Kpn-Nco I fragment (5′ homoloy region) from plasmid #183 was then subcloned into plasmid #196 in front of the 3× FLAG region (plasmid #199). The loxP site in the 5′ region of exon 1 was cloned using annealed primers #5 and #6 and ligated into plasmid #199 at the Xho I and Xba I sites. This gave rise to plasmid #204, which was sequenced to confirm the integrity of the 3xFLAG-LoxP-exon 1 junction. A 1.8 kb Xba I-Sac I fragment including OGR1 exon 1 and ‘3′ untranslated region from plasmid #188 was subcloned into plasmid #203 to generate plasmid #209. A 7.7 kb Hind III fragment of plasmid #208 harboring the neo-cassette flanked by loxP sites and the 3′ OGR1 homology region was subcloned into plasmid #209 to give the OGR1 targeting vector #218. The functionality of LoxP sites were confirmed in an *E. coli* strain BNN123 constitutively expressing recombinant Cre. The sequences of the primers used were:

#1: 5′ GGT ACC GGA GGA CGT GAG CAA CAA CT
#2: 5′ ATG TTC CCC ATG GTT GGG CCA GAA
#3: 5′ TCC TAC TTG GCA GTA CAT CT
#4: 5′ ATC TCG AGC TTG TAC TCG TCA TCC TTG
#5: 5′ TCG AGA TAA CTT CGT ATA GCA TAC ATT ATA CGA AGT TAT
#6: 5′ CTA GAT AAC TTC GTA TAA TGT ATG CTA TAC GAA GTT ATC
#7: 5′ TCT AGA GGG AAC ATC ACT ACA GAA AAC TC
#8: 5′ AGG AAC TTG GCT AAG GAC

The OGR1 targeting constructs were injected into ES cells at the Transgenic Mouse Core Facility at Case Western Research University (Cleveland, OH). Chimeric mice with germ line FLAG-tagged OGR1 genes flanked by two loxP sites were generated. Through breeding to C57/BL6 mice, these mice were used to generate heterozygous mice with one copy of FLAG-tagged OGR1. Subsequent breeding of heterozygous OGR1^fl/+^ mice generated homozygous OGR1^fl/fl^ mice. All offsprings from the breeding of heterozygous OGR1^fl/+^ mice were genotyped by DNA analysis of tail clips. Initially both Southern blot and PCR analyses were conducted to confirm genotype. Subsequent genotyping was mainly conducted using PCR analyses. Homozygous mice with OGR1 floxed (OGR1^fl/fl^ ) were bred to germ line Cre mice Zp-3 (a transgenic mouse line for the inactivation of loxP-flanked target genes specifically in the female germ line [26]; Jackson Labs, Bar Harbor, Maine). In addition, they were bred to Prm (Protamine-Cre, a transgenic mouse line for the inactivation of loxP-flanked target genes specifically in the male germ line of mice [27]; kindly supplied by Dr. Guangbin Luo, Case Western Reserve University) to generate OGR1^+/−^Cre mice, which were bred further to generate OGR1^−/−^ (KO or deficient) mice. The OGR1^fl/fl^ mice were designated OGR1 FL. OGR1^−/−^ mice in the mixed background have been backcrossed to C57/BL6 for 10 generations, the OGR1^+/+^ 10^th^ generation mice were designated OGR1 wild type (OGR1 WT).

### Phenotype analyses

OGR1 KO mice were viable and fertile. A complete mouse phenotype analysis was conducted in two pairs of OGR1 KO and FL mice (8 weeks old, one male and one female in each group) at the Mouse Phenotypes Shared Resource, OHIO State University, Columbus, OH. For determining the adiposity index, a previously published method was used [28].

### Tissue distribution assays

Organs were excised from mice and fixed in Zinc (BD, Franklin Lakes, NJ) for 12–24 hr, then kept in 70% ethanol prior to being embedded in paraffin and then sectioned at 5 µm. Post deparaffinization and rehydration, the slides were treated with citrate buffer for 20 min and with H_2_O_2_ (0.3%) in ethanol for 15 min. Universal horse serum was used to block the tissue followed by anti-FLAG antibody (1∶200 dilutions) treatment. VECTOR NovaRED was used as the substrate and hematoxylin QS as a counter stain.

### RNA isolation, reverse transcription-PCR (RT-PCR)

Tissues were excised from mice and pulverized after snap freezing in liquid nitrogen. Total RNA was extracted using RNeasy mini kit (Qiangen, Maryland) and reverse transcribed by M-MLV (Invitrogen, Carlsbad, CA). Derived cDNAs were amplified using PCR master mix (Promega, Madison, WI). Primer sequences for OGR1 were as follows: 5′-CTCAATGACCTCCTTGTGATTG-3′ and 5′-CTACCAGAAAACTCCTCACTATC-3′. β-actin was amplified as a housekeeping gene with primers 5′-ACCGCTCGTTGCCATTAGTGATGA -3′ and 5′-AAGGCCAACCGTGAAAAGATGACC-3′.

### Brown adipose tissue isolation and proliferation of brown adipose derived cells (BADCs)

Brown fat was dissected from the dorsal aspect of the thorax between the scapulae, and formalin (10%) fixed & then paraffin embedded, followed by H & E staining. To culture BADCs, brown fat was cut into small pieces and placed into same volume of collagenase solution (DMEM, 1% penicillin/streptomycin, 1% BSA, 1 mg/mL collagenase type I, filtered). This suspension was placed in a 37°C shaker at 200 rpm for 60 min. Following this, DMEM medium (10 mL) containing 10% FBS, 1% penicillin/streptomycin, 2% glutamine, 1% non essential amino acids and 1/1000 β-mercaptoethanol was added to the brown fat cells. After centrifugation at 2,000 rpm for 5 min, the white layer was removed and the cell pellets were resuspended into 10 mL culture medium, and filtered through a cell strainer. The cells were then transferred to cell culture dishes and allowed to reach confluence. For the proliferation/survival assays, cells were starved for 24 hr, then treated with different stimulus for 24 and 48 hr [3]. 10 µL of 3-(4,5-Dimethylthiazol-2-yl)-2,5-diphenyltetrazolium bromide (MTT, 5 mg/mL) was added into the cells 3 hr before harvesting. The absorbance of the solution at 550 nm was determined using a Vector^3^ spectrophotometer (PerkinElmer, Waltham, MA).

### Macrophage and osteoclast generation and characterization

For peritoneal macrophages, mice were injected i.p. with 1 mL of TG (4%). Macrophages were collected four days after injection. Cells were harvested by peritoneal washings using RPMI. After washing, the cells were cultured in RPMI with 10% FBS for 2 hr. The non-adherent cells were removed and over 80% of the adherent cells were macrophages [29]. Bone marrow derived macrophages (BMMs) were obtained from the femurs of 6–10-week-old mice [30]. In brief, the ends of the bones were cut off, and the marrow was flushed from amputated femurs using a 1 mL syringe to obtain a suspension, which was then passed through a 27-gauge needle for dispersion. Marrow cell suspensions (500 µL; 1×10^6^ cells) were plated in low M-CSF [3% L 929 cell-conditioned medium (L-CM) as a source of M-CSF] in RPMI containing 20% FBS. L-CM was produced by seeding 2.5×10^5^ cells in a 175-cm^2^ tissue culture flask with 50 mL of basic medium until cells were confluent. The supernatant was then collected, filtered through a 0.2-µm filter, and frozen in aliquots at −80°C. After allowing stroma cells to adhere overnight, non-adherent cells were collected and plated in high M-CSF (30% L-CM). After three days, the medium was removed along with non-attached cells, and new medium containing 30% L-CM was added. Adherent macrophages were homogeneous populations after 7 days of culture. The cells were stained with Diff-quick and counted. To differentiate macrophages into osteoclasts, fresh medium with RANKL (50 ng/mL) was added on the third day and scored three days later by counting TRAP-positive cells.

### Phagocytosis and nitric oxide (NO) production assays

Peritoneal macrophages (1×10^4^ cells/300 µL) were incubated in 48-well plates and treated with latex beads (1×10^6^) for 3 hr. The assays were terminated by washing cells with ice-cold PBS. The cells were then fixed in methanol for 30 min. Phagocytosis was quantified under a fluorescent microscope by counting the number of internalized beads in at least 200 cells, which was recorded as phagocytotic index by the formula: 100×(cell number with one bead+3.5×cell number with 2–5 beads+8×cell number with 6–10 beads+20×cell number with over 10 beads) /total cell numbers. For NO production, peritoneal macrophages (5×10^5^) in 100 µL of RPMI containing 10% FBS were cultured in 96-well plate for 2 hr at 37°C. Cells were treated by replacing with medium (0.5% FBS in 100 µL) and LPS (50 µL of 1 µg/mL) for 18 hr. The levels of NO in the supernatant were assayed by mixing the same volume of supernatants (100 µL) and Griess reagent in a new 96- well plate. The plates were incubated for 15 min at room temperature and read at 570 nm using a plate reader. The concentration of nitrite in the culture supernatant was calculated from a standard curve generated from diluting sodium nitrite in water at a concentration range of 0.01–100 µM.

### Prostaglandin production and COX-2 expression

95% confluent cells in 24-well plates were treated with different pH buffers for 9 hr or 25 hr [31], [32]. The supernatants were collected for prostaglandin analysis using HPLC-mass spectrometry (API-4000, Applied Biosystems). In brief, prostaglandins were extracted from cell supernatants (500 µL in each sample) in the presence of 14∶0 lysophosphatidic acid (LPA, 10 pmol) as an internal standard using chloroform (2 mL), methanol (2 mL), and HCl (6 N, 10 µL). The ions with the m/z at 351 (the parent ion) and 271 (the daughter ion) were used for identification of prostaglandins. A TARGA C18 5 µM, 2.1 mm ID×10 mm TR-0121-C185 (Higgins Analytical, Southborough, MA USA) HPLC column was used, and the mobile phase was MeOH/water/NH_4_OH (90∶10∶0.1, v/v/v), 6 min/sample. The cell pellets were collected for analyzing COX-2 expression.

### Western blot analyses

Macrophages (10^6^) cultured in 6-well plate were rinsed with PBS and stimulated by LPS (100 ng/mL), SPC or LPC (2.5 µM) for 30 min. Cells were lysed in 100 µL of laemmli sample buffer (BIO-RAD, Hercules, CA), extract were resolved on a 10% SDS-polyacrylamide gel and transferred to polyvinylidene difluoride membrane. Membranes were blocked in 5% skim milk for 2 hr and then incubated with indicated primary antibodies overnight at 4°C. After incubation with corresponding secondary antibodies for 1 hr at room temperature, membranes were developed using an ECL plus western blotting detection system (GE Healthcare, Buckinghamshire UK). Protein loading was verified by stripping and reprobing the blots with antibodies against β-actin_._

### Cyclic AMP (cAMP) production and cell survival at different pH

Peritoneal macrophages were plated in 24-well plates, treated with different pH buffers containing the phosphodiesterase inhibitor isobutylmethylxanthine (IBMX, 1mM) for 30 min, and then lysed with 0.1 M HCl (60 µL). Cell lysates were used for the cAMP assay according to the kit instructions (Cyclic AMP EIA Kit, from Cayman, Cat # 581001). For the survival assays, macrophages or bone marrow-derived osteoclasts were plated in 96-well plates for 2 hr at 37°C with 5% CO_2_ and then cultured in medium at different pHs at 37°C with 0% CO_2_ for 20 hr.

### Bone histomorphometrical and immunohistochemical analyses

Bone samples from 8-week-old animals were fixed in Bouin's solution overnight, decalcified in EDTA (14%) for 12 days, and embedded in paraffin. Sections were then stained with Masson's Trichrome Stain Kit for morphological observation.

### Melanoma tumorigenesis

B16-F10 cells were cultured in RPMI with 10% FBS. 1×10^7^ and 5×10^5^ cells in 100 µL of PBS were subcutaneously injected into flanks of the mixed background and the pure C57/BL6 background mice, respectively. The mice were sacrificed when they appeared moribund (9–14 days post-injection). The animal research complied with all relevant federal guidelines and policies of the Laboratory Animal Resource Center at Indiana University School of Medicine.

### Statistical analyses

All data in this study was expressed as mean±SD of three or more independent experiments in triplicate. Differences between treatment groups were analyzed using Student's *t* test and two-tailed distribution in Microsoft excel.

## Results

### Generation of OGR1 deficient mice and OGR1 expression in tissues

**Figure 1:**
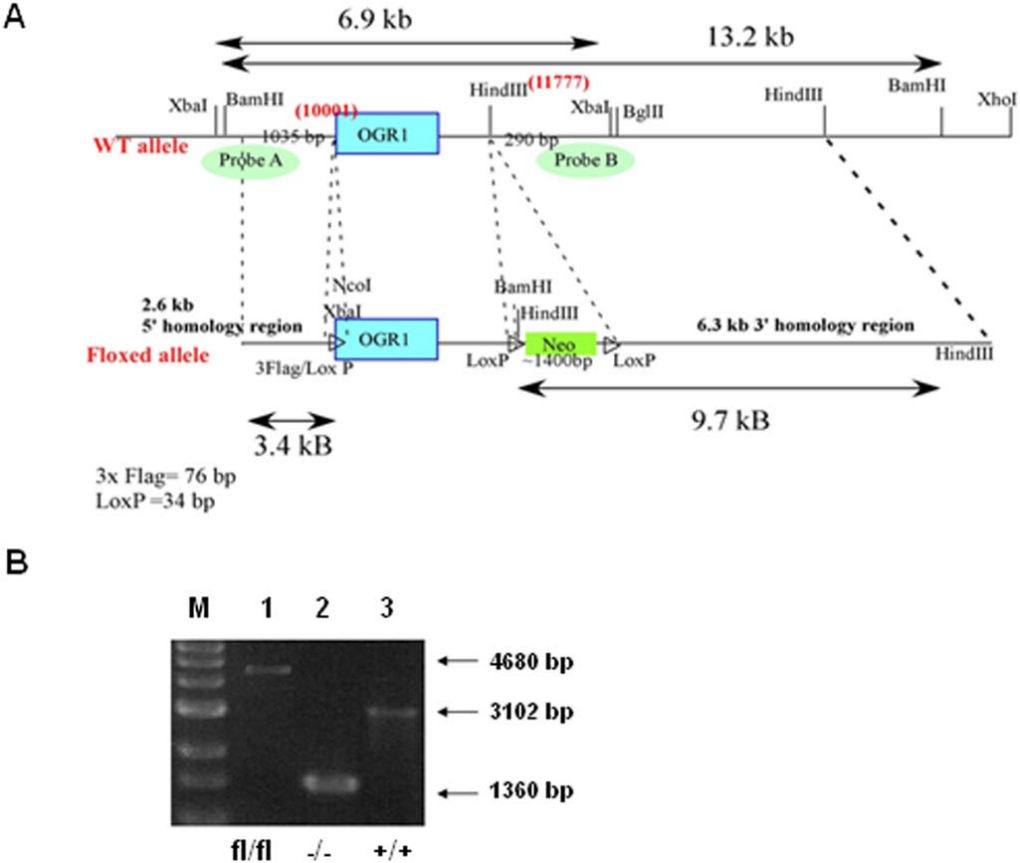
Generation of conditional OGR1 deficient mice. (A) Strategy for constructing the OGR1 targeting vector. (B) Genotyping was performed to identify floxed (FL), −/− (KO) and +/+ (WT) mice.

**Figure 2:**
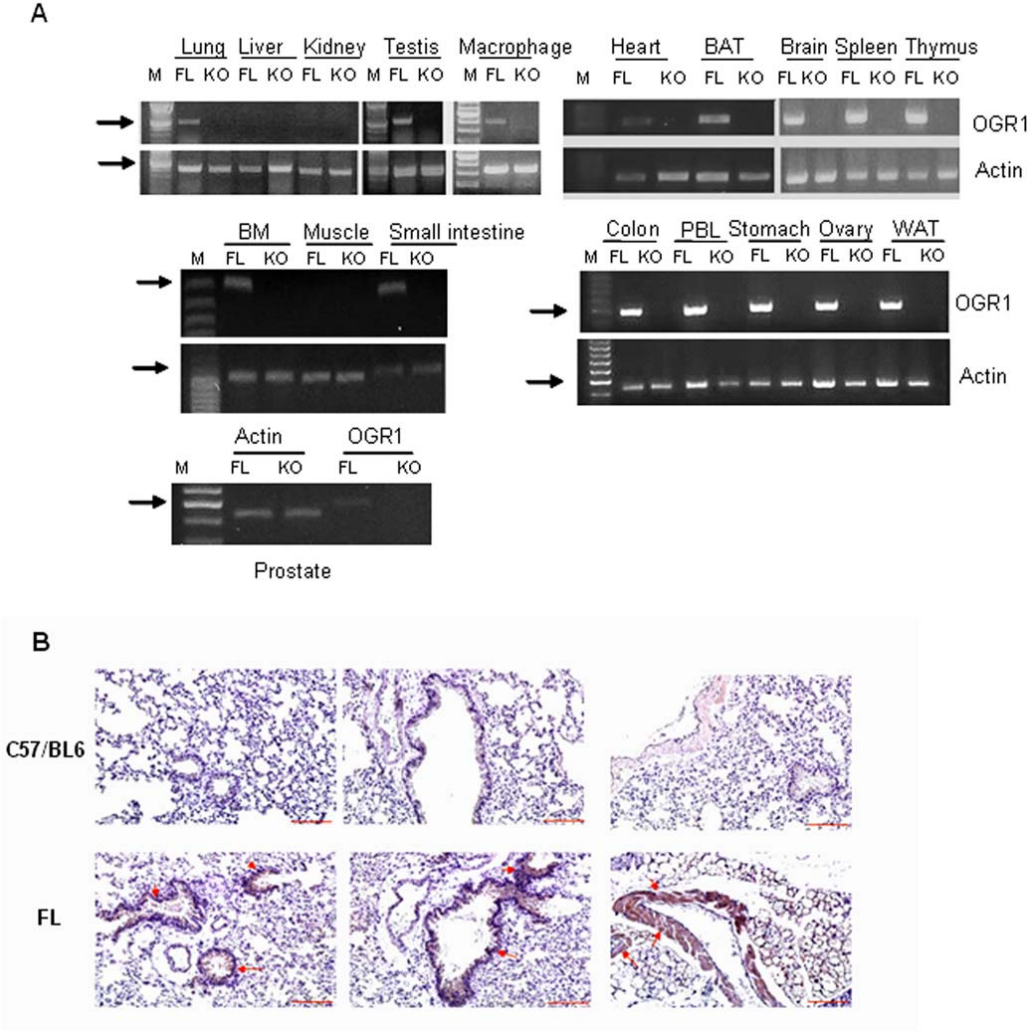
OGR1 expression in mouse tissues and cells. (A) OGR1 expression in various tissues from FL and KO mice detected by RT-PCR. The PCR program was 94°C, 2 min; 25 cycles for actin and 35 cycles for OGR1 (94°C, 30 s; 55°C, 1 min; 72°C, 1 min); 72°C, 10 min, except both actin and OGR1 in macrophages were amplified by 30 cycles. BAT, brown adipose tissue; BM, bone marrow; PBL, peripheral blood leucocytes. The arrows indicate DNA ladders with 500 bp. (B) OGR1 distribution in lung tissue from FL and C57/BL6 mice. Anti-FLAG M2 antibody (1∶200) was used. Representative positively stained cells are indicated by arrows. Scale bar = 100 µm.

To study the physiological role of OGR1, we have established an OGR1 deficient mouse strain. Generation of OGR1 deficient mice was described in detail in the Materials and Methods. The overall strategy for constructing the OGR1 targeting vector was shown in Figure 1A. The results from OGR1 genotyping were shown in Figure 1B. OGR1 was expressed in the lung, testis, heart, brain, spleen, thymus, brown fat, small intestine, colon, peripheral blood leukocytes (PBL), macrophages, stomach, ovary, and white fat, but not in the liver, kidney, and skeletal muscle of the OGR1 FL mice, as detected by RT-PCR. OGR1 expression in the prostate was weak, but detectable (Figure 2A). The complete lack of OGR1 expression in OGR1 KO mice was confirmed in all tissues examined. We inserted a three-FLAG tag in front of OGR1 in the knock-in construct so that the endogenous OGR1 expression at the protein level can be detected in tissues using anti-FLAG M2 antibody. Immunohistochemical staining of the lung of OGR1 FL mice showed that OGR1 was expressed in the cuboidal/columar epithelial cells covering the large airways of the lung and in the vascular smooth muscle cells surrounding a large blood vessel (Figure 2B). This tissue expression pattern of OGR1 was similar to that observed in human tissues as demonstrated previously [1], [33].

### General physiological analyses of OGR1 KO mice and brown adipose tissue (BAT) abnormality in the mixed background

**Figure 3:**
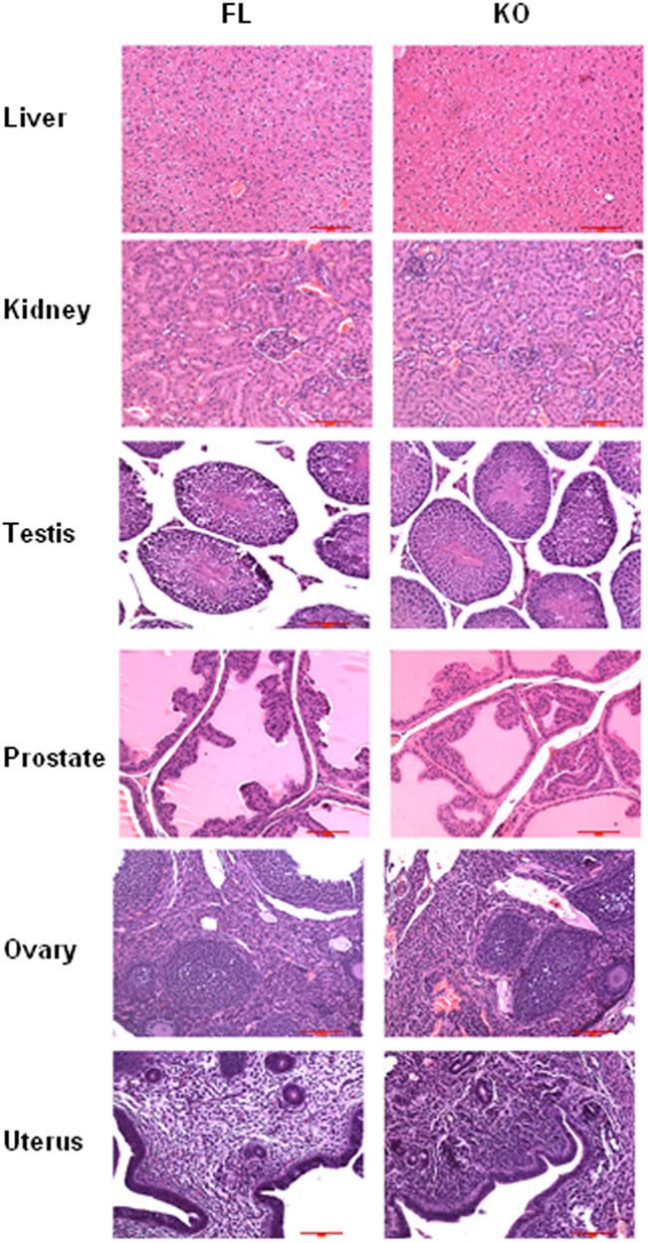
Immunohistochemistry of FL and KO mice tissues. The representative H & E staining of the liver, kidney, testis, prostate, ovary and uterus were presented. Scale bar = 100 µm.

OGR1 KO mice were viable and fertile. A complete mouse phenotype analysis was conducted in two pairs of OGR1 KO and OGR1 FL mice (8 weeks old, one male and one female in each group). Body weights did not differ substantially among the mice examined. In these two pairs of mice, the spleens of OGR1 KO mice appeared to be somewhat smaller than those of the FL mice, whereas the livers and hearts of the KO mice were somewhat larger than those of the FL mice. However, additional analysis in more mice did not demonstrate statistical differences in these organ weights (data not shown). Histological analyses in FL and KO did not reveal significant differences in the majority of tissues, and representative data are shown in Figure 3.

**Figure 4:**
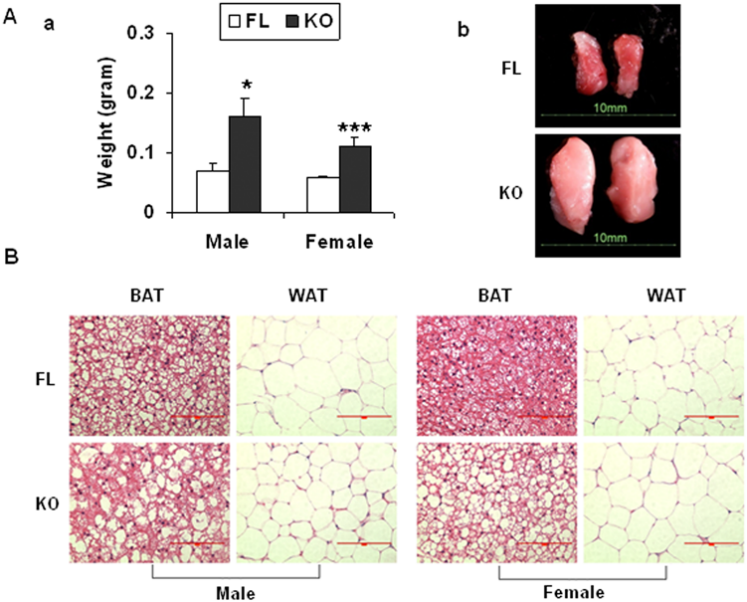
Brown adipose tissue abnormality in OGR1 KO mice in the mixed background. (A) Brown adipose tissues in the KO mice were larger than those in FL mice. (a) Weights of BAT (9 mice from FL and 9 mice from KO). (b) Representative appearance of BAT from FL and KO mice. (B) H & E staining of BAT and WAT in FL and KO mice. * represents a *P* value of <0.05, and *** represents a *P* value of <0.001. Scale bar = 100 µm.

The initial analyses also revealed that the intrascapular BAT deposit was heavier in the KO (0.35 and 0.28 g) than the FL (0.10 and 0.11 g) mice, which may be related to altered adiposity in the KO mice. Consistent differences in BAT weights and sizes were observed in additional pairs of FL and KO mice (Figure 4A). H & E staining showed that while the white fat tissues (WAT) from FL and KO mice were similar, the BAT from KO mice were less dense when compared to those from FL mice (Figure 4B), which may be related, in part, to the enlarged size of BAT in OGR1 KO mice.

**Figure 5:**
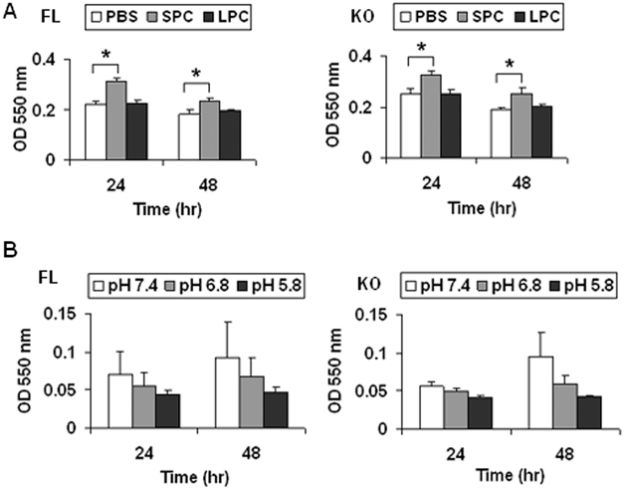
Proliferation of brown adipose tissue-derived cells in OGR1 KO mice in the mixed background. (A) SPC stimulated proliferation was not significantly different in BAT from FL *vs.* KO mice. SPC (2.5 µM) or LPC (2.5 µM) was used. (B) The effect of pH on proliferation was not significantly different in FL *vs.* KO. The results in this figure are representative of at least 3 independent experiments. * represents a *P* value of <0.05.

OGR1 has been shown to have proton-sensing activity in various cells [3], [6], [9], [34]. In addition, SPC may modulate OGR1's actions [6], [9]. SPC has been shown to induce proliferation of human adipose tissue-derived mesenchymal stem cells via activation of c-Jun N-terminal kinase (JNK) [35]. To test whether BAT from FL and KO mice respond differentially to pH and/or SPC, we isolated BADCs and tested the effects of SPC or pH changes on cell proliferation. SPC (2.5 µM) modestly stimulated cell proliferation at 24 hr and this effect was not significantly different in BADCs from FL versus KO mice (Figure 5A). Acidic pH induced lower levels of proliferation than the physiological pH (7.4), and again no statistical difference was observed in BADCs from FL *vs.* KO mice (Figure 5B). We have backcrossed OGR1 deficient mice into C57/BL6 background (10 generations). When we examined BAT in WT and KO mice in the C57/BL6 background, no difference was observed (data not shown), suggesting that the BAT abnormality is related to the mouse background.

### OGR1 deficiency reduced macrophage production in mice of the mixed background

**Figure 6:**
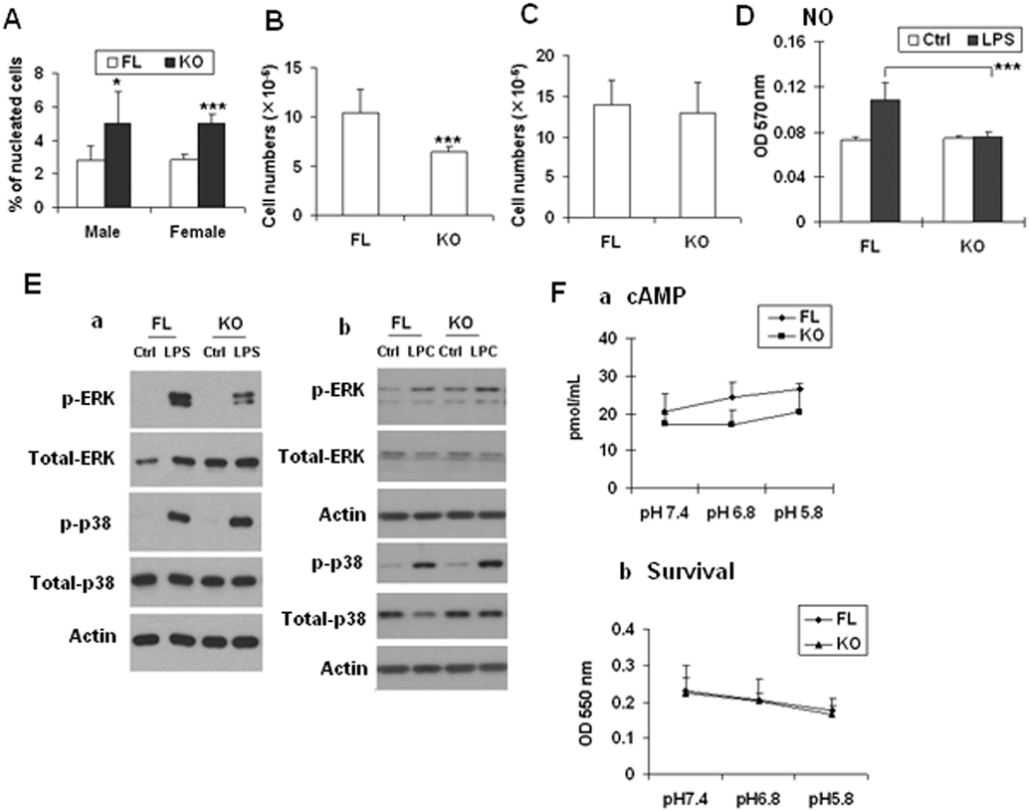
Reduction of TG-induced peritoneal macrophage numbers and NO production in OGR1 KO mice in the mixed background. (A) The percentages of monocytes in peripheral blood were higher in KO than that in WT mice. Blood samples were obtained from facial vein of FL and KO mice (n = 6 in each group). (B) TG-stimulated peritoneal macrophage numbers in KO mice were reduced when compared to those from WT mice (n = 20 in each group). (C) Bone marrow-derived macrophage numbers in FL and KO mice after M-CSF stimulation (n = 6 in each group) were not significantly different. (D) Nitric Oxide production from KO peritoneal macrophages was reduced than that of WT macrophages. Macrophages were stimulated with LPS (330 ng/mL) for 18 hr. (E) LPS-induced ERK activation was reduced in OGR1 KO macrophages. TG-induced peritoneal macrophages were treated with LPS (100 µg/mL) (a), or LPC (2.5 µM) (b) for 30 min prior to sample collection for Western blot analyses. (F) pH-modulated cAMP formation (a) or cell survival (b) was not significantly different in peritoneal macrophages from FL and KO mice. Cells were treated with indicated pH buffer and IBMX (1 mM) for 30 min. Proton effect on the survival of macrophages were conducted for 20 hr. The results in this figure are representative of at least 3 independent experiments. * represents a *P* value of <0.05, and *** represents a *P* value of <0.001.

Microscopic examination of the tissues did not reveal obvious genotype-related differences between KO and FL mice. Hematological tests, including packed cell volume (PCV), hemoglobin (g/dL), red blood cells (RBC, ×10^12^), mean corpuscular or cell volume (MCV, fL), mean corpuscular or cell hemoglobin concentration (MCHC, g/dL), red cell distribution width (RDW), RBC morphology, mean platelet volume (MPV), nucleated cells (×10^9^/L), neutrophils (%), lymphocytes (%), monocytes (%), eosinophils (%), basophils (%), leukocyte morphology, and platelet count were not significantly different between KO and FL mice (data not shown), except that the percentage of monocytes and neutrophils appear to be higher and lower, respectively, in OGR1 deficient mice. We have repeated blood cell counts in more pairs of mice and confirmed that the percentage of monocytes in blood was higher in OGR1 KO mice versus FL mice (Figure 6A). OGR1 was expressed in mouse macrophages (Figure 2A). Interestingly, KO mice produced less peritoneal macrophages in response to TG, when compared to the FL mice (Figure 6B). However, when BMMs were counted after treating bone marrow cells with M-CSF, no difference was observed in KO and FL mice (Figure 6C), although OGR1 was also expressed in bone marrow cells and PBL (Figure 2A). These results suggest that the reduced peritoneal macrophages was not related to deficiencies in bone marrow cells or macrophage precursor cells in the blood, and it may be more specific to TG and/or bacterium-induced process.

We further tested whether macrophages from KO mice have altered phagocytotic activity. We found that the phagocytosis indexes in FL and KO mice were 27.5±5.8 and 26.6 ±4.4, respectively, which were not statistically different (more than 10 pairs of mice were tested). LPS-induced NO production in macrophages is important for their biological function [36]. We found that NO production stimulated by LPS was significantly reduced in OGR1 KO mice compared to FL mice (Figure 6D). LPS can also activate several signaling pathways, including ERK and p38 MAP kinase pathways. While LPS-induced p38 activation or LPC-induced ERK and p38 phosphorylation were not significantly different, LPS-stimulated phosphorylation of ERK was significantly reduced in macrophages from KO mice when compared to that from FL mice (Figure 6E). To address the proton sensing issue, we tested cAMP production and cell survival of macrophages in response to pH changes. Statistical analysis indicated that there was no significant difference in these activities at any of the pHs tested between FL and KO macrophages (Figure 6F).

Similar to BAT abnormality, we found that TG-stimulated peritoneal macrophage number were not significantly different in mice in a pure C57/BL6 background, suggesting this is also a mouse background-dependent phenotype.

### The role of OGR1 in osteoclastogenesis

**Figure 7:**
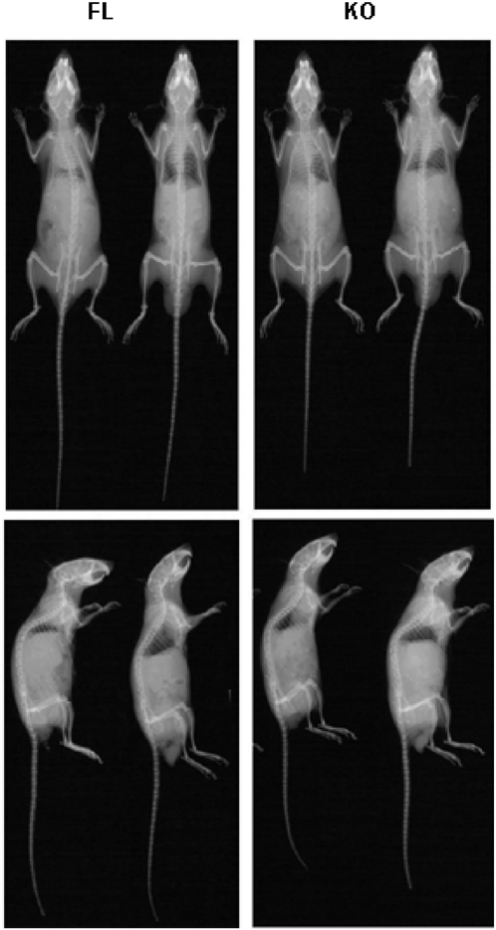
Role of OGR1 in osteoclastogenesis of the mixed and the C57/BL6 background mice. Radiographs of WT (left) and KO (right) mice (conducted in Mouse Phenotyping Shared Resource, Ohio State University).

**Figure 8:**
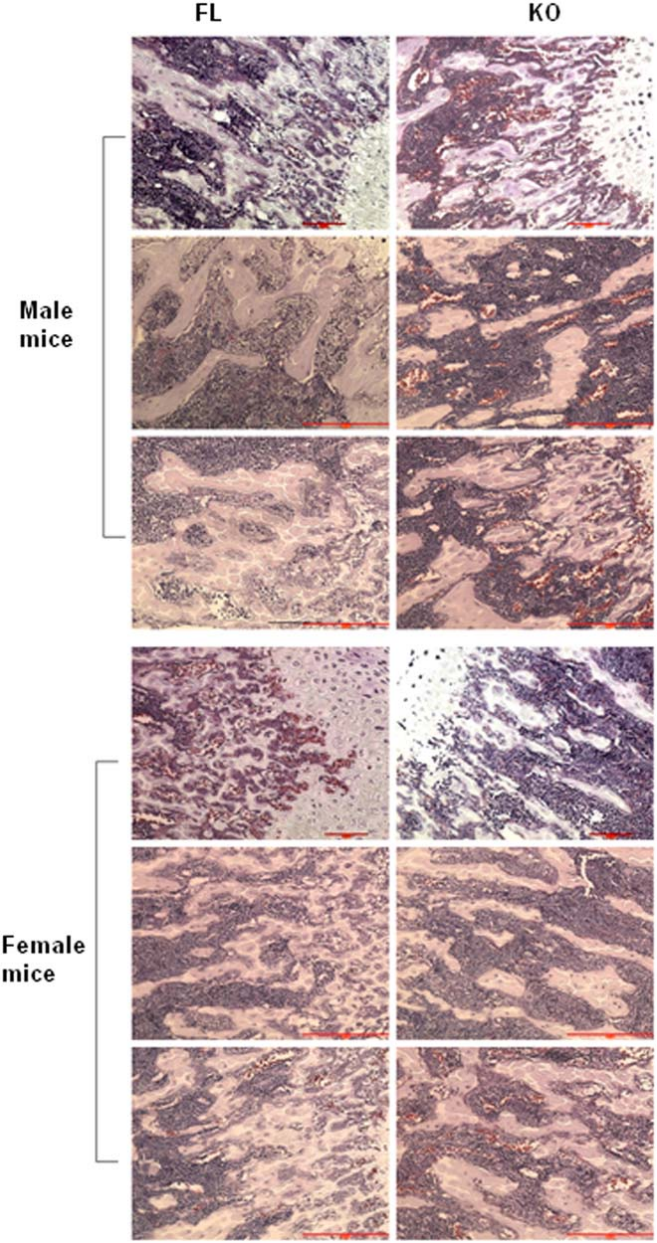
Histology of femurs from FL and KO mice. Masson's Trichrome staining of bones in male and female OGR1 FL and KO mice. Nuclei were stained black, cytoplasm was red, muscle fibers were red, and collagen was blue. Scale bar = 100 µm.

OGR1 has been implicated in osteoclastogenesis [21], [22], [37]. OGR1 deficient mice are the best system to test this concept under physiological conditions. Gross abnormalities of KO mouse bones were not observed as shown in the X-ray of bone from both FL and KO mice (Figure 7), suggesting that even OGR1 might even have a role in osteoclastogenesis, the overall effects are not apparent and that a redundant role of other genes may be involved *in vivo*. This is further supported by bone immunohistochemical analyses using Masson's Trichrome staining. The histomorphometry showed that the bone volume/total volume, osteoblasts number, osteoclasts number, trabecular thickness and bone-marrow cavity were not significantly different between OGR1 KO and WT mice in either mixed background or pure C57/BL6 background (Figure 8).

**Figure 9:**
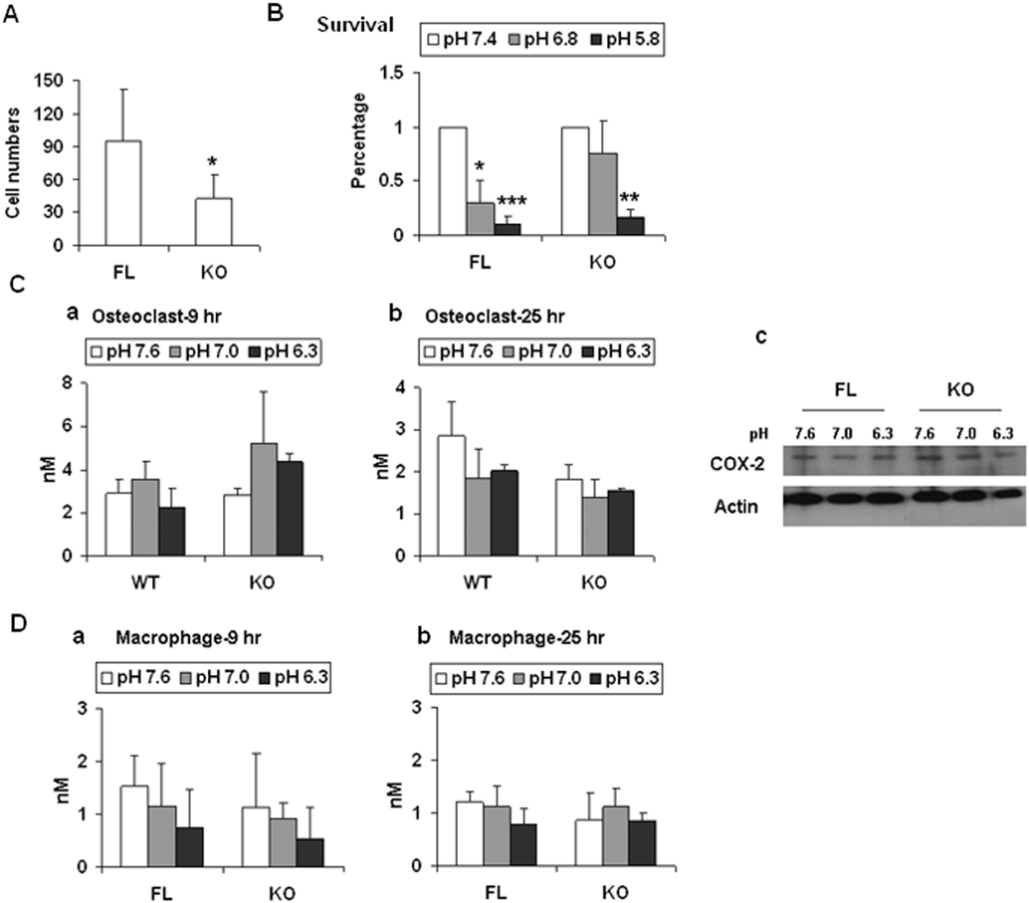
Reduced osteoclasts differentiated from bone marrow cells in OGR1 KO mice. (A) M-CSF and RANKL-induced osteoclast numbers in OGR1 KO mice were reduced. (B) pH effects on survival of osteoclasts. Osteoclasts were cultured in medium with different pH for 20 hr. (C) Prostaglandin production and COX-2 expression in osteoclasts. Osteoclasts were cultured in different pH buffer (300 µL) in 24-well plates for indicated times and the supernatants were collected for prostaglandin analysis (a and b) and the cells were lysed for COX-2 expression analysis at 9 hr (c). The differences in different groups are not statistically significant (*P*>0.05). (D) Prostaglandin production in macrophages. The results in this figure are representative of at least 3 independent experiments. * represents a *P* value of <0.05, ** represents a *P* value of <0.005, and *** represents a *P* value of <0.001.

To test osteoclastogenesis more specifically, isolated bone marrow cells were induced to differentiate by M-CSF and RANKL and the numbers of osteoclasts were counted. After testing more than 20 pairs of mice, a consistent reduction in osteoclast formation was observed in KO mice in both the mixed and the pure C57/BL6 backgrounds (Figure 9A). Therefore, this is a phenotype of OGR1 that was persistent in the C57/BL6 background. We also examined the survival of osteoclasts at different pHs. While OGR1 FL mice had significantly reduced cell survival at pH 6.8, when compared to survival at pH 7.4, the OGR1 KO mice had a similar survival rate at pH 7.4 and 6.8 (Figure 9B). This was the only pH-dependent difference detected in cells from FL and OGR1 KO mice in the mixed background, which was also detected between cells from WT and OGR1 KO mice in the C57/BL6 background.

It has been reported that *in vitro* OGR1 is involved in prostaglandin production and activation of COX-2 in bone and smooth muscle cells in response to pH changes [31], [32]. We tested these concepts in osteoclasts and macrophages. We found that neither prostaglandin production from either osteoclasts (Figure 9C, a and b) or macrophages (Figure 9D, a and b) nor COX-2 expression in osteoclasts (Figure 9C, c) was differentially regulated in FL and KO mice. This suggests that the effects observed *in vitro* using overexpression systems may not completely mimic *in vivo* physiological systems, and/or that other OGR1 subfamily genes, such as G2A, GPR4, and/or TDAG8 may play similar and redundant roles *in vivo*.

### OGR1 deficiency reduced tumorigenesis of melanoma cells in both mixed and C57/BL6 backgrounds

**Figure 10:**
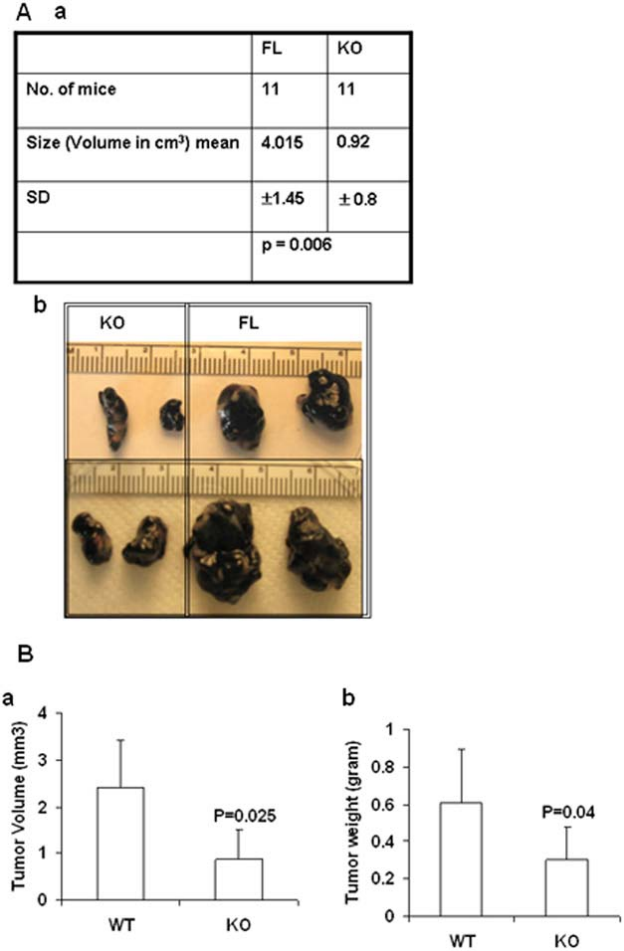
Melanoma growth was suppressed in OGR1 KO mice in both mixed and C57/BL6 backgrounds. (A, a) Tumorigenesis of melanoma cells was reduced in OGR1 KO mice in the mixed background. B16-F10 cells (10^7^ mouse melanoma cells) were injected s.c into FL and KO mice in the mixed background. Mice were sacrificed at 9–14 days post-injection. (b) Representative pictures of the tumors developed in FL and KO mice in the mixed background. (B). Summary of tumor volumes (a) and weights (b) in mice with the C57/BL6 background (n = 10 in WT and n = 12 in KO). Tumorigenesis of melanoma cells was reduced in OGR1 KO mice.

**Figure 11:**
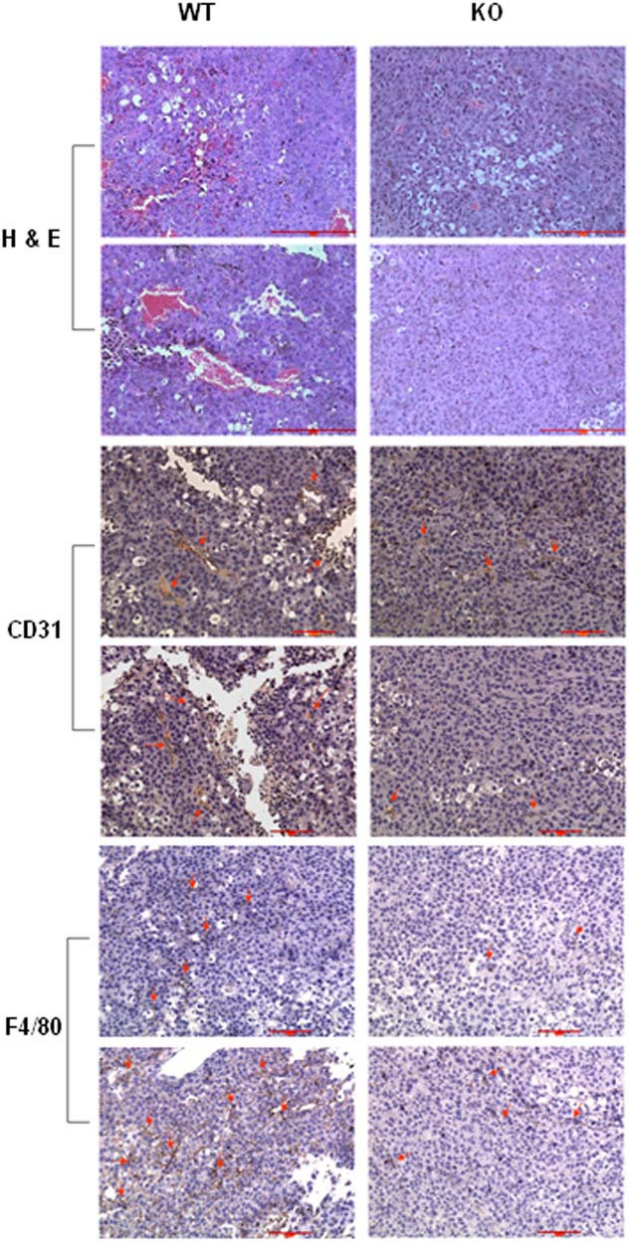
Immunohistochemistry staining of melanoma tissue from WT and KO mice. H & E staining of tumor sections for gross observation. Staining of tumor section with anti-CD31 (1∶50) for angiogenesis analysis. Staining of tumor section with anti-F4/80 (1∶75) to view macrophage infiltration. Representative positively stained cells were indicated by arrows. Scale bar = 100 µm.

Since macrophages play important roles in cancers, the abnormalities in TG-stimulated macrophages prompted us to test whether host cell OGR1 deficiency affects tumor development in mice. We used melanoma B16-F10 cells, since these cells are highly malignant and when used in relatively high numbers, they form tumors even in non-syngenic mice. We found that the tumor sizes were significantly smaller in OGR1 KO mice when compared to WT mice in either the mixed (Figure 10A) or the pure C57/BL6 background (Figure 10B). H & E staining and CD31 staining showed that the tumors from KO mice had less blood vessels and CD31-positive endothelial cells than those from WT mice, suggesting reduced angiogenesis in tumors in KO mice. In addition, less tumor-infiltrated F4/80-positive macrophages were detected in tumors from KO mice (Figure 11), suggesting that decreased TAMs may be also responsible for reduced tumorigenesis.

## Discussion

### Limited proton-sensing activities were observed in cells from OGR1-deficient mice

The proton sensing activities of the OGR1 subfamily of GPCRs, including G2A, GPR4, and TDAG8 have been reported previously (6, 8, 15, 18, 24, 32). Using gene knockout techniques, mice deficient in G2A, GPR4, and TDAG8 have been previously reported [10], [11], [12]. In certain, but not all, cellular and biological assays, pH-sensing activities have been detected in GPR4- and TDAG8-, but not in G2A-deficient cells [8], [10], [20]. A common scenario that has been revealed from these studies is that the pH-dependent activities are highly cell type-, signaling pathway-, and biological activity-specific. We have tested the pH-induced effects in macrophages, osteoclasts, and BADCs, the three major cell types where some alterations have been observed in the OGR1 KO mice. However, these studies showed that there was only a very limited pH-induced cellular effect (osteoclast cell survival) was observed. While this manuscript was in preparation, Mogi *et al* have reported that TDAG8, but not OGR1 is involved in the pH induced inhibitory effect on TNF-α production in macrophages [20], which is consistent with our results. The most likely explanation of these results is that other OGR1 subfamily GPCRs are also expressed in these tested cells and they play certain redundant roles *in vivo*. These questions can only be further clarified when double, triple or even quadruple knockout mice are generated. This subfamily of GPCRs may also be regulated or modulated by a group of lysophospholipids (11, 25, 27, 36, 38). Similar to the pH effects, we did not observed altered lipid effects in WT verses KO cells, which can be interpreted as a redundant effect or lack of lipid effect through OGR1 in these cells.

Taken together, OGR1 subfamily receptors mediate the effects of and/or are regulated by extracellular pH changes and certain lysophospholipids. These effects are highly cell type-, G protein-, signaling pathway-specific. The systems used to detect these effects are also highly relevant. In particular, the conclusions derived on the functions/signaling pathways of this family of GPCRs from *in vitro* over-expression systems are not completely consistent with the results obtained from knockout mice as described above, emphasizing the importance to study the pathophysiological roles of these receptors *in vivo* and the potential redundant functions among OGR1 subfamily GPCRs.

### The potential involvement of OGR1 in BAT and macrophages were mouse genetic background-dependent

We have found that the cell types affected by OGR1 in the mixed or the C57/BL6 background are all potentially derived from mesenchymal stem cells (MSCs) or hematopoietic stem cells from bone marrow, which are capable of differentiating into monocytes, osteoclasts, osteoblasts, and adipocytes, among other cell phenotypes. We consistently found higher accumulations of BAT both in terms of dimension and weight in OGR1 KO mice in the mixed background. However, these differences disappeared when mice were in the C57/BL6 background, suggesting that a modifying gene(s) in different mouse background may play a role in regulating BAT. To our knowledge, OGR1 is the first gene showing an affect on the size of BAT in a mouse-background dependent manner. The mechanisms by which OGR1 regulates this phenotype and the identification of the modifying gene warrant further studies. The extent of brown fat abnormality observed in OGR1 KO mice did not clearly affected the mouse physiological function in these mice under normal living conditions. It might be interesting to test whether under different temperatures these mice behave differently from WT mice, since brown fat is mainly involved in thermogenesis.

The role of OGR1 in macrophages may be related to its function in tumorigenesis of melanoma cells, since reduced TAMs were observed in tumors from KO mice, which also correlated with reduced tumor sizes. However, similar to the BAT phenotype, altered peritoneal macrophages were not consistently observed in mice with a pure C57/BL6 background, suggesting that a modifying gene(s) is likely to be involved.

### OGR1 was likely to be involved in osteoclastogenesis and tumorigenesis

The phenotypes of OGR1 KO mice related to osteoclast and melanoma tumor formation were consistent in both mixed and C57/BL6 backgrounds. Our results suggested that although OGR1 may play a role in osteoclastogenesis, its effect on overall bone physiology was rather minimal. It is possible that under certain pathological conditions, the defect in osteoclast numbers and/or their response to pH changes will affect some biological functions. This is true when we test tumorigenesis when mice were challenged with melanoma cells in either the mixed and or the C57/BL6 background. The mechanisms by which host cells OGR1 acts to regulate tumorigenesis remains to be further investigated. Intriguingly, we have recently shown that OGR1 over-expression in tumor cells displays a tumor metastasis suppressing role for prostate cancer [2]. It is highly interesting to further study the apparent opposing roles of OGR1 in tumor *vs.* host cells.

In summary, similar to other members of this family, OGR1 deficiency did not significantly affect overall mouse physiology. While OGR1 deficiency did not result in a strong phenotype by itself, it may generate interesting and strong phenotypes when mice are challenged, as we report here for melanoma cells. In addition, OGR1 and its subfamily GPCRs may have redundant roles *in vivo*, which can be further revealed when more than one of these genes is depleted. With this report, the initial phenotypic analyses of the four members of OGR1-subfamily GPCRs has now been completed and more interesting results are expected to be generated from double to quadruple deficient mice.
