# Correction: Abnormalities in Osteoclastogenesis and Decreased Tumorigenesis in Mice Deficient for Ovarian Cancer G Protein-Coupled Receptor 1

**DOI:** 10.1371/journal.pone.0125463

**Published:** 2015-04-13

**Authors:** Hui Li, Dongmei Wang, Lisam Shanjukumar Singh, Michael Berk, Haiyan Tan, Zhenwen Zhao, Rosemary Steinmetz, Kashif Kirmani, Gang Wei, Yan Xu

In Fig 2A of the original published article, the bands from some samples that were run on a single gel, but were not adjacent in the original gel, were spliced together. A revised figure in which separated lanes are represented by gaps between gel images is provided here. Specifically, the DNA ladders shown for the Testis and Macrophage data were not run in adjacent lanes to each sample (top row, middle panel of the original published figure). PBL and Stomach samples were not run in adjacent lanes to each other (middle row, right panel of the original published figure); a lane showing bands from pancreas samples was removed from this gel due to low levels of the actin control. Additionally, the published middle left panel showing BM, Muscle, and Small Intestine data was upside down. Review by Indiana University Office of Research Integrity found that these alterations did not change the interpretation of the data or the study results. The original raw files for the gel images are provided as a supplementary file in addition to the revised figure. The authors apologize for the errors and any inconvenience that it may have caused.

**Fig 2:**
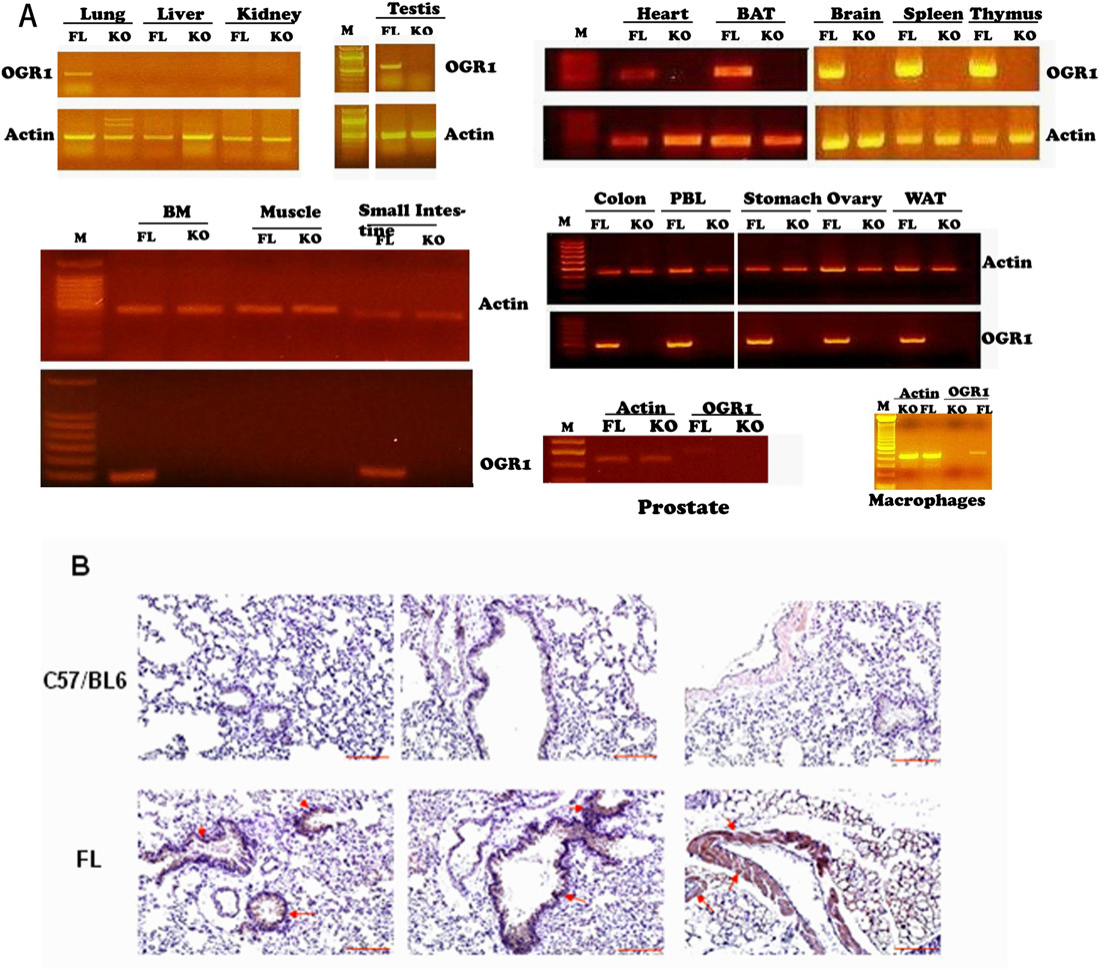
OGR1 expression in mouse tissues and cells. (A) OGR1 expression in various tissues from FL and KO mice detected by RT-PCR. The PCR program was 94°C, 2 min; 25 cycles for actin and 35 cycles for OGR1 (94°C, 30 s; 55°C, 1 min; 72°C, 1 min); 72°C, 10 min, except both actin and OGR1 in macrophages were amplified by 30 cycles. BAT, brown adipose tissue; BM, bone marrow; PBL, peripheral blood leucocytes. The arrows indicate DNA ladders with 500 bp. (B) OGR1 distribution in lung tissue from FL and C57/BL6 mice. Anti-FLAG M2 antibody (1:200) was used. Representative positively stained cells are indicated by arrows. Scale bar = 100 μm.

## Supporting Information

S1 File Raw files. This file contains:
Image file for gel showing BM, Muscle, and Small Intestine samples, employed to generate the revised figure.
Image file for gel showing Macrophage samples, employed to generate the revised figure.
Image file containing gel showing Testis samples, employed to generate the revised figure.
Image file for gel showing Brain, Spleen, and Thymus samples, employed to generate the revised figure.
Image file for gel showing Colon, PBL, Stomach, Ovary, and WAT samples, employed to generate the revised figure.
(ZIP)
