# Supplementary figures and images for: Correction: Abnormalities in Osteoclastogenesis and Decreased Tumorigenesis in Mice Deficient for Ovarian Cancer G Protein-Coupled Receptor 1
Source: PLoS One. 2015 Apr 13;10(4):e0125463. doi: 10.1371/journal.pone.0125463 (PMC4395281; doi:10.1371/journal.pone.0125463)

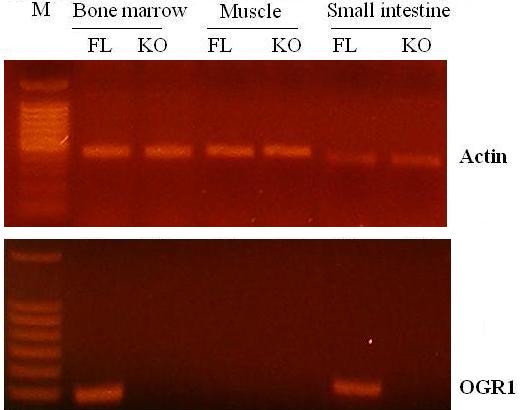

Supplement: S1 File — This file contains: Image file for gel showing BM, Muscle, and Small Intestine samples, employed to generate the revised figure. Image file for gel showing Macrophage samples, employed to generate the revised figure. Image file containing gel showing Testis samples, employed to generate the revised figure. Image file for gel showing Brain, Spleen, and Thymus samples, employed to generate the revised figure. Image file for gel showing Colon, PBL, Stomach, Ovary, and WAT samples, employed to generate the revised figure. (ZIP) [file pone.0125463.s001.zip › bone marrow-muscle-prostate-small intestine edt.JPG]

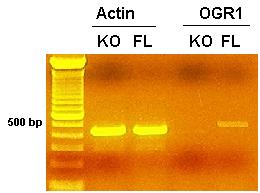

Supplement: S1 File — This file contains: Image file for gel showing BM, Muscle, and Small Intestine samples, employed to generate the revised figure. Image file for gel showing Macrophage samples, employed to generate the revised figure. Image file containing gel showing Testis samples, employed to generate the revised figure. Image file for gel showing Brain, Spleen, and Thymus samples, employed to generate the revised figure. Image file for gel showing Colon, PBL, Stomach, Ovary, and WAT samples, employed to generate the revised figure. (ZIP) [file pone.0125463.s001.zip › OGR1 RT in macrophages.JPG]

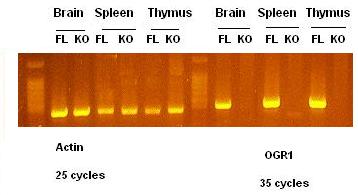

Supplement: S1 File — This file contains: Image file for gel showing BM, Muscle, and Small Intestine samples, employed to generate the revised figure. Image file for gel showing Macrophage samples, employed to generate the revised figure. Image file containing gel showing Testis samples, employed to generate the revised figure. Image file for gel showing Brain, Spleen, and Thymus samples, employed to generate the revised figure. Image file for gel showing Colon, PBL, Stomach, Ovary, and WAT samples, employed to generate the revised figure. (ZIP) [file pone.0125463.s001.zip › spleen and thymus-ed.JPG]

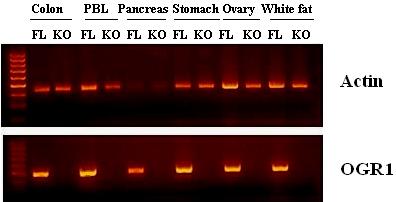

Supplement: S1 File — This file contains: Image file for gel showing BM, Muscle, and Small Intestine samples, employed to generate the revised figure. Image file for gel showing Macrophage samples, employed to generate the revised figure. Image file containing gel showing Testis samples, employed to generate the revised figure. Image file for gel showing Brain, Spleen, and Thymus samples, employed to generate the revised figure. Image file for gel showing Colon, PBL, Stomach, Ovary, and WAT samples, employed to generate the revised figure. (ZIP) [file pone.0125463.s001.zip › STOMACH COLON FAT.JPG]

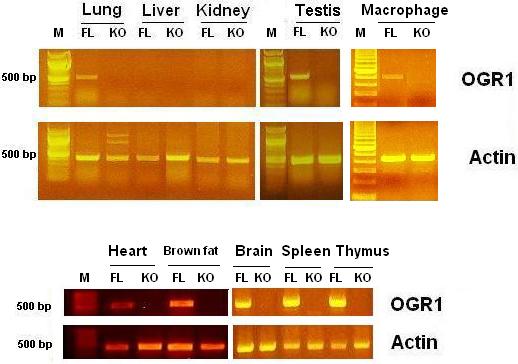

Supplement: S1 File — This file contains: Image file for gel showing BM, Muscle, and Small Intestine samples, employed to generate the revised figure. Image file for gel showing Macrophage samples, employed to generate the revised figure. Image file containing gel showing Testis samples, employed to generate the revised figure. Image file for gel showing Brain, Spleen, and Thymus samples, employed to generate the revised figure. Image file for gel showing Colon, PBL, Stomach, Ovary, and WAT samples, employed to generate the revised figure. (ZIP) [file pone.0125463.s001.zip › TISSIE _LEGEND.JPG]
